# Potential utility of miRNAs for liquid biopsy in breast cancer

**DOI:** 10.3389/fonc.2022.940314

**Published:** 2022-08-04

**Authors:** Xiangrong Liu, Dimitri Papukashvili, Zhixiang Wang, Yan Liu, Xiaoxia Chen, Jianrong Li, Zhiyuan Li, Linjie Hu, Zheng Li, Nino Rcheulishvili, Xiaoqing Lu, Jinfeng Ma

**Affiliations:** ^1^ Shanxi Province Cancer Hospital/Shanxi Hospital Affiliated to Cancer Hospital, Chinese Academy of Medical Sciences/Cancer Hospital Affiliated to Shanxi Medical University, Taiyuan, China; ^2^ Department of Pharmacology, School of Medicine, Southern University of Science and Technology, Shenzhen, China

**Keywords:** liquid biopsy, breast cancer, miRNA, biomarker, biofluids

## Abstract

Breast cancer (BC) remains the most prevalent malignancy due to its incidence rate, recurrence, and metastasis in women. Conventional strategies of cancer detection– mammography and tissue biopsy lack the capacity to detect the complete cancer genomic landscape. Besides, they often give false- positive or negative results. The presence of this and other disadvantages such as invasiveness, high-cost, and side effects necessitates developing new strategies to overcome the BC burden. Liquid biopsy (LB) has been brought to the fore owing to its early detection, screening, prognosis, simplicity of the technique, and efficient monitoring. Remarkably, microRNAs (miRNAs)– gene expression regulators seem to play a major role as biomarkers detected in the samples of LB. Particularly, miR-21 and miR-155 among other possible candidates seem to serve as favorable biomarkers in the diagnosis and prognosis of BC. Hence, this review will assess the potential utility of miRNAs as biomarkers and will highlight certain promising candidates for the LB approach in the diagnosis and management of BC that may optimize the patient outcome.

## Introduction

**Figure 1:**
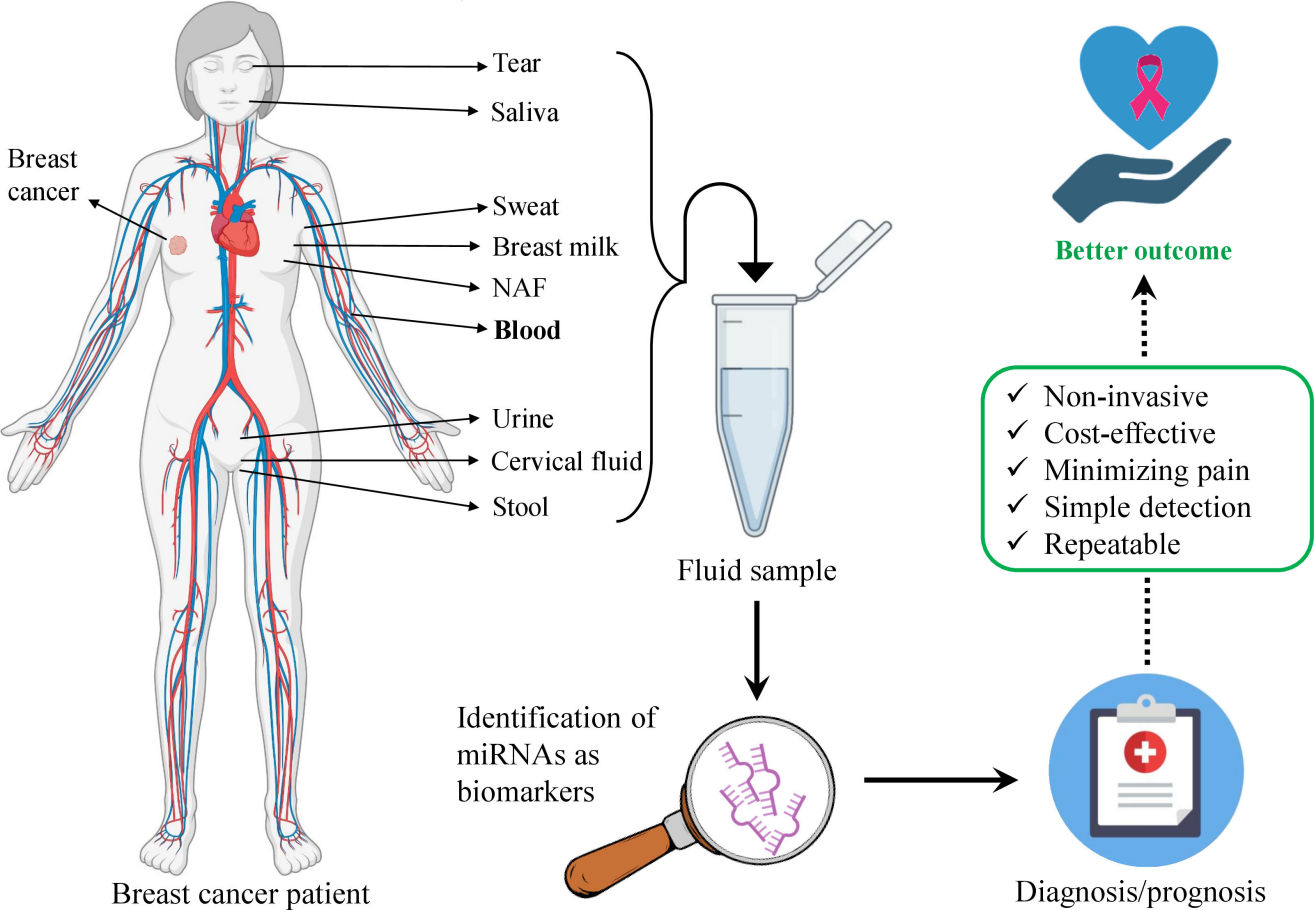
Schematic illustration of body fluids-based miRNA utility as biomarkers in breast cancer diagnosis and prognosis. The body fluids are the source of miRNAs that can be obtained *via* liquid biopsy. Bold-type indicates the most promising and studied body fluid for detecting miRNAs in breast cancer. Several listed biofluids have not yet been studied for miRNA detection as biomarkers in breast cancer, still, they represent potential liquid biopsy candidates.

Breast cancer (BC) is the most malignant and emergent tumor among women cancer patients (1, 2). Millions of women are diagnosed with BC and over half a million deaths are reported annually (3, 4). There are four main molecular subtypes of BC– luminal A, luminal B, human epidermal growth factor receptor 2 (Her2)-enriched, and basal-like (5). The first two subtypes usually have a more favorable prognosis. The most common subtype- luminal A is characterized by the expression of estrogen and progesterone receptors (ER/PR) while the luminal B subtype is additionally characterized by the absence of Her2. In the Her2-positive subtype, the Her2 gene is highly expressed and the cell proliferation rate is also high. Basal-like subtype does not express any of the mentioned markers and accounts for up to 20%. Additionally, there is one more subtype– normal-like subtype which represents the rarest BC and accounts for only up to 10% of all BCs. The normal-like subtype is characterized by the expression of ER, PR, and Her2 and clinically it is between basal-like and luminal A subtypes (6). The early diagnosis and advancements in treatment are the main focus of BC research. Mammography and tissue biopsy remain the standard screening methods until now (7). However, a number of disadvantages exist. E.g., the false-positive result of mammography requires additional analysis that may lead to potential side effects (8) while the eventual result may be negative. Except for the mentioned, mammography is related to exposure to ionizing radiation (9). Mammography imaging often is not sufficient for evaluation and requires further analysis. It is often performed along with tissue biopsy. This requires the imaging to be done before and after the biopsy to ensure the accuracy of sampling and biopsy marker placement. This technique is advantageous for its specificity to the suspicious tissue which is detected on a mammogram. Nonetheless, the disadvantages include a painful, long time of the procedure, invasiveness, and high-cost (10). Moreover, it is not adequately comprehensive to obtain the complete landscape of BC (11). Fortunately, a relatively new approach liquid biopsy (LB) that is a non-invasive and simple technique compared with surgical biopsies enables obtaining the important information of tumor *via* simple body fluids-based samples, mainly blood. The other advantages comprise the short time of the procedure, precise and real-time results, serial sampling, and monitoring. This enhances its application for the early diagnosis that enables better management of BC, better outcome, and, most importantly, less mortality (11). Indeed, the application of LBs has revolutionized the existing standard clinical approach and may play a critical role in diagnosing and monitoring the tumor as well as the response to the treatment. LB next-generation sequencing (NGS)-based FoundationOne Liquid CDx test was approved by Food and Drug Administration (FDA) in 2020 for application for diagnosis *via* detecting multiple tumor biomarkers in plasma (12). LB is undoubtedly considered one of the most perspective detection approaches for many cancers including BC. Indeed, LB allows the detection of circulating tumor components such as cancer cells, RNA, or circulating tumor DNA (ctDNA) in liquid specimens (13). Despite their low concentrations in the liquid samples, they still can be used as indicators and biomarkers of cancer. This characteristic makes LB a sensitive, advanced, alternative, reliable, and cost-effective approach for the diagnosis and screening of BC (14). Remarkably microRNAs (miRNAs) detected in LB specimens seem to be promising measurable indicators of BC. The change in their levels reflects the various conditions of the body. miRNAs are small non-coding RNAs that serve as gene-regulator molecules in the body. As miRNAs are implicated in various signaling pathways, alteration of their levels indicates certain conditions, e.g., various cancers (11, 15–17). Indeed, proof-of-concept studies demonstrated that the composition of miRNAs and their abundance in the blood are altered in cancer patients (11, 18, 19). Additionally, Li et al. have studied miRNAs in BC patients and demonstrated that 13 miRNAs were found to be differentially expressed in patients with metastatic BC (20). Kim et al. successfully monitored the expression levels of miR-21 and miR-155 in the urinary samples of mice injected with BC cells (21). Hence, in this review, we summarize recent and relevant data on circulating miRNAs (c-miRNAs) for their potential role in the diagnostics of BC to become a part of the LB approach in clinics. A schematic illustration of the potential utility of miRNAs detected in body fluids as biomarkers for BC diagnosis and prognosis is given in **Figure 1**.

## miRNA role in cancers

Since the identification of miRNA in 1993, it is gaining growing attention. Currently, over 3000 miRNAs are found in almost all human body fluids. These miRNAs are implicated in various processes and play a significant role in the progression or regression of a number of diseases (22–26). miRNAs vary in their function as they have a strong capacity of regulating multiple gene expression. miRNAs have a significant impact on tumorigenesis, apoptosis, embryogenesis, cell proliferation, and differentiation among many other biological processes (27). miRNAs regulate gene expression *via* the post-transcriptional mechanism (6). The biogenesis of miRNA consists of several steps and takes place in the nucleus and cytoplasm of the cell. First, the primary miRNA (pri-miRNA) is transcribed mostly from introns into the nucleus and cleaved by endonuclease Drosha which results in the formation of precursor miRNA (pre-miRNA). Pre-miRNA is then translocated into the cytoplasm by Exportin 5 (XPO5) where it undergoes further processing and the mature miRNA duplex is formed. Argonaute (AGO) protein family recognizes miRNA and leads to the loading of miRNA guide strand into the RNA-induced silencing complex (RISC). The passenger strand is degraded and approximately 22 nucleotide-length miRNA containing seed region is ready to bind the specific region of the target messenger RNA (mRNA) molecule on the 3’untranslated region (3’UTR) that consequently leads to the silencing of the target gene (22, 28). Remarkably, miRNAs are implicated in the regulation of key pathways that are usually genetically altered in various cancers. As cancer represents a disease when the normal cells become abnormal *via* acquiring the ability of uncontrollable growth and division, miRNAs that are regulators of cell proliferation, differentiation, and apoptosis, play a crucial role in this condition. Indeed, several signaling pathways have been identified to be actively involved in cancers– RTK/RAS/MAP pathway, phosphatidylinositol 3-kinase (PI3K)/protein kinase B (AKT) (29), Wingless (WNT)/β-catenin (30), etc.

### OncomiRs, tumor suppressors

According to the role in cancer, miRNAs elicit either oncogenic or tumor-suppressive functions *via* silencing target genes. Therefore, they may be considered oncomiRs or tumor suppressor miRNAs (6). miRNAs that are associated with cancer due to their upregulated levels are called oncomiRs (31, 32). Interestingly, tumor angiogenesis is the formation of new blood vessels that helps the tumor to grow *via* supplying with the blood (33) and plays a key role in cancer development. It is a complex process and comprises activation, migration, proliferation, and differentiation of the endothelial cell. There are numerous factors that control each of these steps positively or negatively (34). A number of miRNAs may act as pro-angiogenic or anti-angiogenic factors. Evidently, upregulation of miR-155 increases proliferation, invasion, migration, and tube formation in triple-negative BC (TNBC) and other cancers *via* targeting tumor suppressor von Hippel-Lindau (VHL) (35). TNBC is a type of BC with no commonly found BC receptors– PR, ER, and Her2. Elevated miR-210 expression increases the endothelial cell migration and its levels in BC represent a prognostic factor (34, 36). miR-182 promotes invasion and proliferation *via* inducing vascular endothelial growth factor A (VEGF-A) in BC (37). miR-10b is the first miRNA with oncogenic action that was discovered in metastatic BC cells. Its expression levels are positively correlated with metastasis and invasion as the action of miR-10b induces overexpression of RAS homolog family member C (RHOC) *via* inhibiting homeobox D10 (HOXD10) mRNA translation (38). Another miRNA miR-21 also influences the oncogenic mechanism *via* inhibiting tumor suppressor genes. It promotes the growth and metastasis of tumor cells. Most importantly, miR-21 is the most commonly found oncomiR in BC. Its upregulation is directly correlated with the poor prognosis of BC (38, 39) and angiogenesis (34, 40). The significance of miR-21 is conditioned by a number of target genes that are affected by miR-21 (38). On the contrary, certain miRNAs act like tumor suppressors inhibiting tumor cell growth and preventing metastasis. miR-335 is one of the tumor suppressor miRNAs that inhibits tumor growth *via* inducing apoptosis (41). miR-16, miR-26a, and miR-101, among a number of other miRNAs, are reported to elicit tumor-suppressive properties in BC (15).

### Role of miRNAs in breast cancer

A number of miRNAs have been reported to have a regulatory function in the pathways implicated in the development of BC. Indeed, certain expression levels of certain miRNAs, e.g., miR-222, are increased in the serum of patients with BC (6). Hannafon et al. have demonstrated remarkably high concentrations of miR-21 and miR-1246 in exosomes obtained from plasma of BC patients compared with healthy controls (42). Si et al. have assessed the impact of miR-21 on tumorigenesis *via* transfecting anti-miR-21 antagomiR into the MCF-7 BC cells and demonstrated that the cell growth was inhibited *via* augmented apoptosis in MCF-7 cells as well as in xenograft mouse model. Interestingly, the decreased cell proliferation was associated with the downregulation of B-cell lymphoma 2 (Bcl-2) protein (43). Zhao et al. observed a similar outcome after miR-21 knockdown *via* antagomiR-21 in a mouse breast tumor model. Moreover, the results revealed that anti-miR-21 suppressed angiogenesis *via* inhibiting HIF-1A/VEGF/VEGFR2-associated signaling pathway (44). Wang et al. have demonstrated the augmented levels of miR-21 in BC patients compared with patients with benign breast tumors and healthy subjects while significantly reduced levels of miR-21 were found in the patients after surgery. Moreover, inhibition of this miRNA impeded tumor progression in BC. Remarkably, leucine zipper transcription factor-like 1 (LZTFL1) was found to be a direct target of miR-21 (45). Therefore, miR-21 may be considered an important pro-angiogenic miRNA (46). miRNAs such as the cluster miR-17-92 are evidenced to be implicated in BC angiogenesis and are called oncomiR-1 (33). miRNAs involved in the angiogenesis of cancers are listed in **Table 1**. There is a number of other miRNAs implicated in various stages of BC metastasis. These miRNAs include miR-9, miR-10a, miR-10b, miR-93, miR-125b, miR-155, miR-181d-5p, miR-191, miR-200, miR-205, miR-221/222, miR-374a, miR-375, miR-378e, etc. (31). There are anti-angiogenic miRNAs that act as tumor suppressors, e.g., miR-7, miR-29b, miR34a, miR124, miR153, miR141, miR-148a, miR-152-3p, miR-205, miR-497, etc. (55). miR-155 is found to be abnormally overexpressed in BC tissues and is associated with advanced tumor stages as well as metastasis (56). On the contrary, miR-497 is found to diminish the tumor development and formation of endothelial cell tube in BC and it was found to be downregulated in BC (57). miR-497 could be considered anti-angiogenic miRNA. Hence, a number of miRNAs are important components of BC angiogenesis which also makes them potential therapeutic targets (55). miRNAs induce tumor progression or suppression *via* regulating gene expression implicated in certain signaling pathways. For instance, miRNAs of the miR-200a-c family represent tumor suppressor miRNAs. miR-200a is found to be under-expressed in BC while its increased levels interfere with WNT/β-catenin signaling which is one of the leading pathways implicated in tumor cell proliferation (58). Pathways including PI3K/AKT, GAS6/MERTK, RTK/RAS/MAP-kinase, and TGF-β signaling are being affected by certain miRNAs that leads to the regulation of tumor progression or suppression (58). According to the abovementioned, there are a number of miRNAs that can be used as potential therapeutic targets for BC. miRNAs involved in inhibiting angiogenesis *via* targeting pro-angiogenic genes are listed in **Table 2**.

**Table 1:** Pro-angiogenic miRNAs in breast cancer and their target genes.

miRNA	Target gene name	Predicted binding site in 3’UTR(miRNA seq bottom, target gene seq top)	miRNA expression level in BC	Ref.
miR-9	*CDH1*	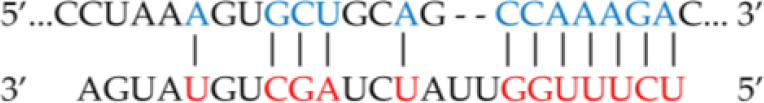	↑	(47)
miR-10b	*HOXD10*	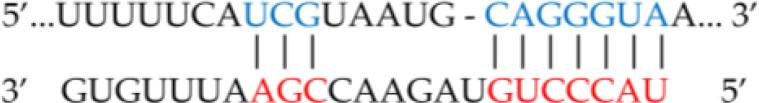	↑	(48)
miR-20a	*VEGFA*	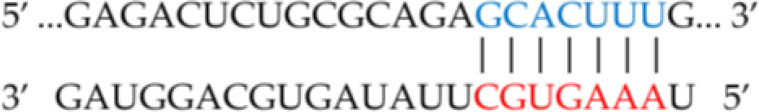	↑	(33)
miR-20b	*HIF-1A*	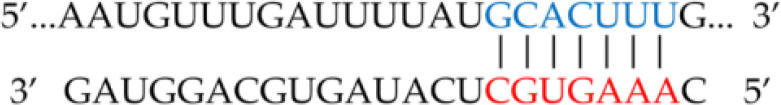	N/A	(49)
	*STAT3*	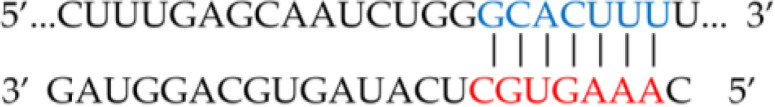	N/A	(49)
	*PTEN*	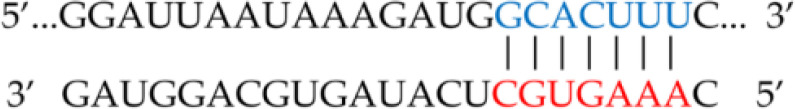	↑	(50)
miR-27a	*FOXO1*	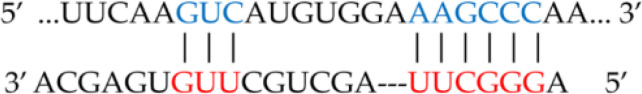	N/A	(51)
miR-93	*LATS2*	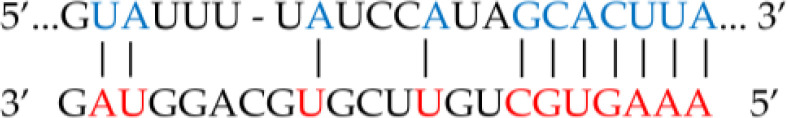	↑	(52)
miR-96	*FOXO1*	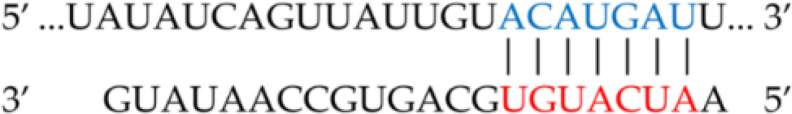	N/A	(51)
miR-155	*VHL*	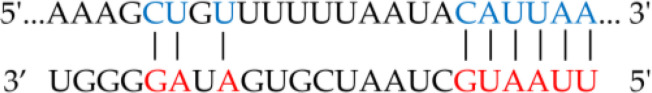	↑	(35, 53)
miR-182	*FOXO1*	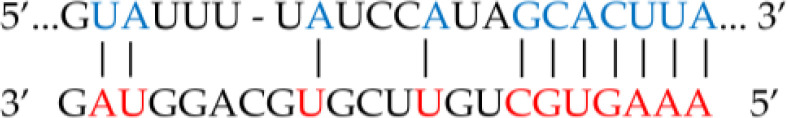	N/A	(51)
miR-210	*EFNA3*	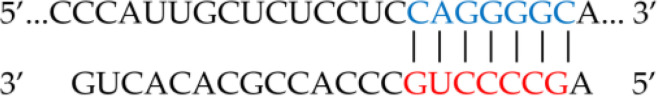	↑	(54)

CDH1, cadherin 1; HOXD10, homeobox D10; VEGFA, vascular endothelial growth factor A; HIF-1A, hypoxia inducible factor 1 subunit alpha; STAT3, signal transducer and activator of transcription 3; PTEN, phosphatase and tensin homolog; FOXO1, forkhead box O1; VHL, von Hippel-Lindau tumor suppressor; EFNA3, ephrin A3.

↑, upregulated level of miRNAs; N/A, not applicable.

**Table 2:** Anti-angiogenic miRNAs in breast cancer and their target genes.

miRNA	Target gene name	Predicted binding site in 3’UTR(miRNA seq bottom, target gene seq top)	miRNA expression level in BC	Ref.
miR-29b	*AKT3*	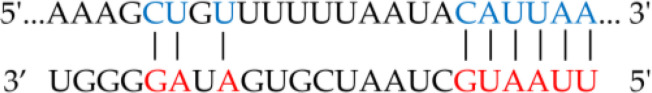	↓	(59)
miR-148a	*AKT3*	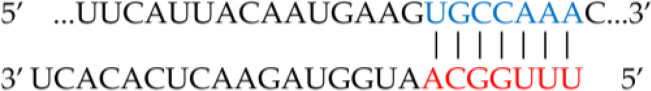	↓	(60)
miR-190	*STC2*	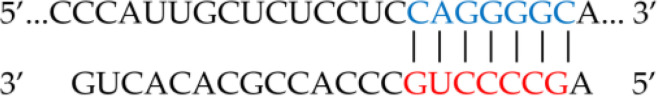	↓	(61)
miR-199b	*ALK1*	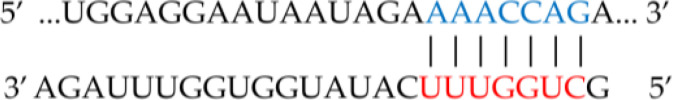	↓	(62)
miR-542	*ANGPT2*	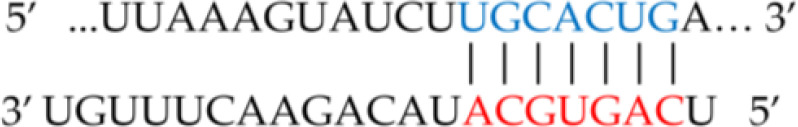	↓	(63)
miR-568	*NFAT5*	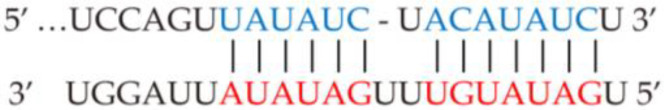	N/A	(64)
miR-4500	*PARP2*	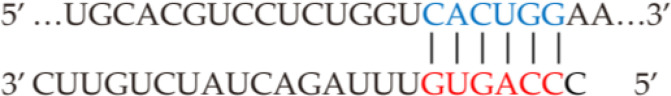	↓	(65)

AKT3, AKT serine/threonine kinase 3; STC2, stanniocalcin 2; ALK1, Activin A receptor like type 1; ANGPT2, angiopoietin 2; NFAT5, nuclear factor of activated T cells 5; PARP2, poly(ADP-ribose) polymerase 2.

↓, downregulated level of miRNAs; N/A, not applicable.

## Liquid biopsy in breast cancer

**Figure 2:**
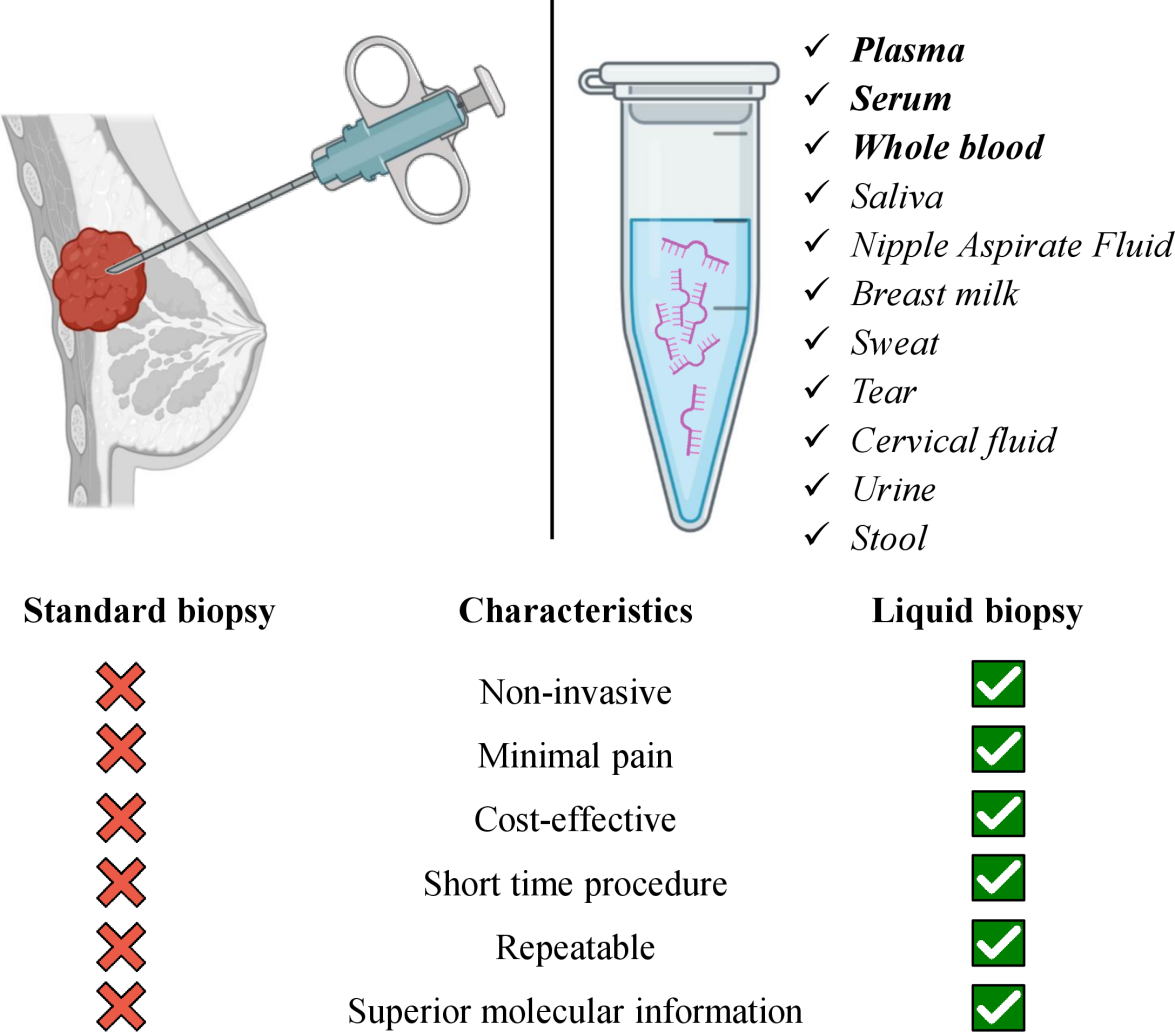
Advantages of liquid biopsy utility in breast cancer management *via* using various samples of body fluids. The biofluids written in bold indicate the most studied samples for miRNA detection in breast cancer. Several listed biofluids have not yet been studied for miRNA detection as biomarkers in breast cancer, still, they represent potential liquid biopsy candidates.

The main barrier to advancing BC treatment is the complex heterogeneity of breast tumors (66). For BC diagnosis, a solid biopsy is a well-established technique in clinics that provides information about tumor histology and subtype. Nonetheless, mishaps in the detection of alternative biomarkers, insufficient tissue obtention, or incompatibility with long-term monitoring, among other limitations still exist (5). Compared with solid biopsy, LB is a more comprehensive diagnostic test that uses blood, urine (67), saliva, and other biological fluids (68, 69). After the sampling circulating tumor nucleic acids and cells are extracted and further analyzed (68, 70). The major tumor component that is usually extracted for the identification of tumor characteristics is circulating tumor cells (CTCs) (71). Except for the CTCs, cell-free DNA (cfDNA), ctDNA (72, 73), and c-miRNAs among other circulating non-coding RNAs (74) can be used as LB markers (13). The advancement of NGS and digital genomic approaches made biomarkers such as c-RNAs, circulating extracellular vesicles, tumor-educated platelets, proteins, etc. possible to detect in biofluids (71, 75, 76). Detection and identification of specific biomarkers are essential for the early diagnosis of BC and other cancers as they can provide additional insight into diagnosis, prognosis, and the response to treatment. In order to achieve this, LB represents a favorable approach. Additionally, in LB, invasiveness and residual disease are minimized that also enables and simplifies the longitudinal monitoring of BC patients. LB potentiates obtaining the complete molecular picture of the tumor and, thus, enhances more precise clinical decisions (5, 77). Indeed, a number of studies successfully consolidate the significance of LB in BC (78–81) and other cancers too (82, 83). Biomarkers detected *via* LB in biofluid samples provide an attractive alternative to biomarkers identified in tissues. The number of body fluids and the existence of all the mentioned potential biomarkers in it as tumor components warrant their promising utility in BC diagnosis, prognosis, and discrimination of different BCs. E.g., serum biomarkers are especially attractive because of the simplicity of sample collection (13). The schematic illustration of the importance and feasibility of LB in BC diagnosis and prognosis is given in **Figure 2**.

## Diagnostic and prognostic function of miRNAs in breast cancer

**Figure 3:**
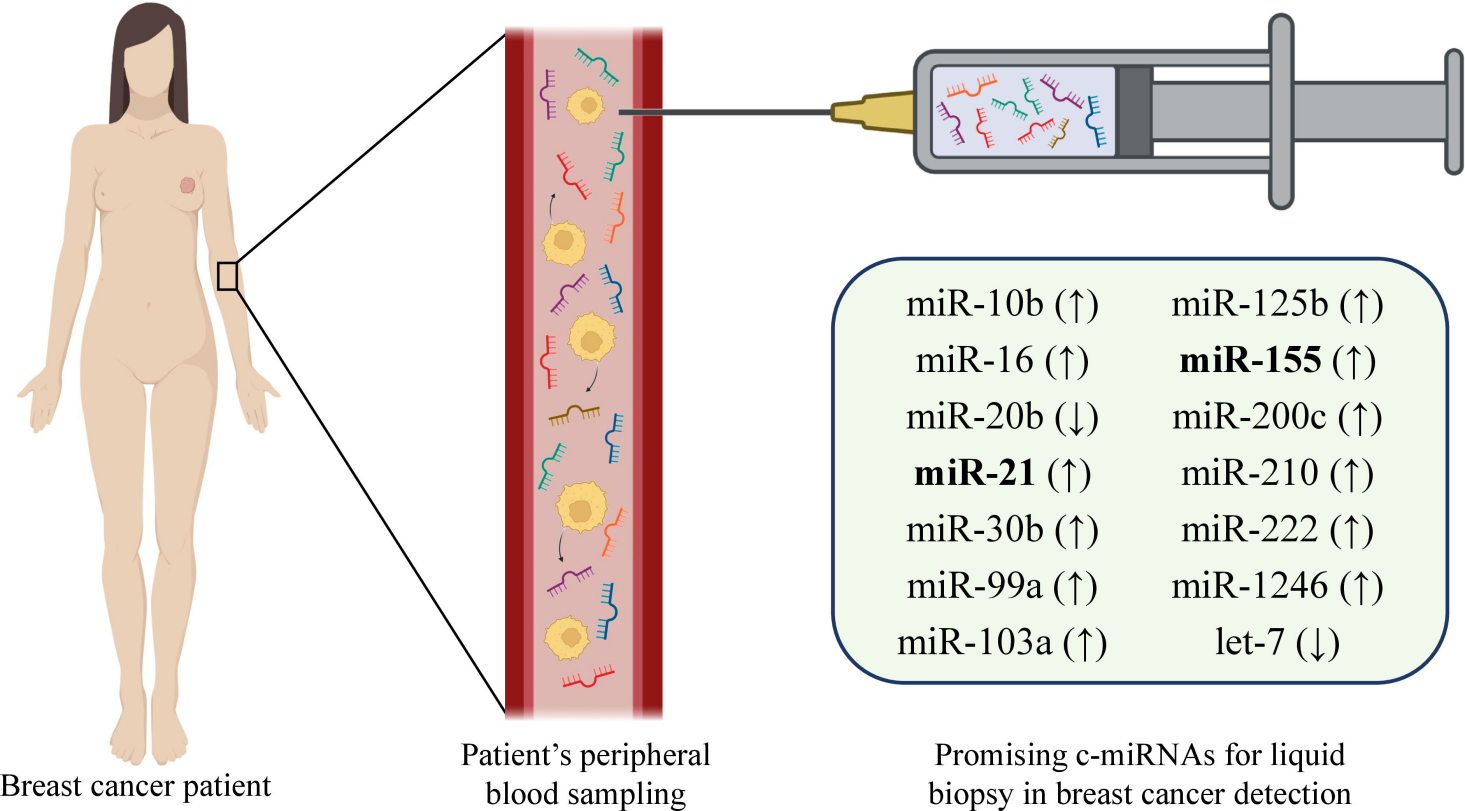
Representative miRNA candidates with the potential of biomarker function in breast cancer diagnosis and prognosis.

Current biomarkers for BC such as CA15.3 and BR27.29 lack enough sensitivity which necessitates seeking the new biomarkers (84). miR-19a, miR-21, miR-24, miR-155, and miR-181b, are found to be overexpressed in patients with BC and downregulated after the surgery or treatment. This also exacerbates the concept of miRNAs’ application as a biomarker for BC diagnosis (45, 84). Henaghan et al. have studied the miRNA expression in the serum of patients with various cancers. The study revealed that certain miRNAs were specific to certain cancer types. E.g., miR-195 was abnormally increased in patients with BC (85). The downregulation of miR-329 in serum and tissue of patients with BC is associated with metastasis to lymph nodes while upregulation of miR-200 is correlated with metastasis to the brain, liver, and lung (84). Indeed, there are a number of studies that demonstrate the potency of certain miRNA utilization as BC biomarkers in various samples (56, 86–93). Apparently, miRNAs maintain the healthy balance of signaling pathways for the normal function of the body. The dysregulation of their normal expression may lead to the progression of the BC. This makes the c-miRNAs attractive biomarker candidates. Application of miRNAs as non-invasive biomarkers in BC allows not only timely diagnosis and successful monitoring of the disease (38), but it also has a discriminatory capacity for, e.g., metastatic and non-metastatic cancers (75). Moreover, in some cases, the miRNA panel demonstrates better discrimination (75, 94). Many individual miRNAs or miRNA combinations associated with certain tumors have been successfully detected in various biofluids such as uterine (95) and cervical (96, 97) fluids, serum, bile (98, 99), etc. Song et al. have screened breast milk for cfDNA and miRNAs and demonstrated that both of these molecules are stably present in breast milk. As they are derived from breast cells they might serve as biomarkers sampled non-invasively (100). Interestingly, levels of miR-155 which is strongly associated with BC were increased in urine samples of BC patients while the expression levels of other miRNAs– miR-21, miR-125b, and miR-451 were significantly lower in the urine samples compared with healthy controls (101). Interestingly, nipple aspirate fluid (NAF) as one of the best samples for detecting miRNAs deserves attention. NAF is an intraductal mammary physiological fluid and represents a source of many biomarkers including miRNAs (102). A number of advantages including its obtainability in the majority of women, the origin of BC, simplicity, and non-invasiveness of sampling, make it a valuable source of miRNAs and LB target in BC diagnosis (102, 103). There are other body fluids where miRNAs have been detected and associated with certain cancers or disorders– saliva (104–106), sweat (107), tear (108–111), and stool (112, 113). Besides, fingernails are also found to be a promising sample for the detection of miRNAs (114). Importantly, the characteristics such as the area under curve (AUC), sensitivity, and specificity should be considered while selecting the most suitable biomarker for BC diagnosis. AUC evaluates the diagnostic capability of studied candidate miRNA in BC detection. When the AUC is close to one, the diagnostic value of miRNA is higher as there are substantial differences between BC and negative control samples. Additionally, while assessing the miRNA diagnostic potential, sensitivity, and specificity that indicate the accuracy of biomarkers also need to be considered (115, 116). E.g., a study revealed that four plasma miRNA panel comprising miR-24, miR-206, miR-373, and miR-1246 could discriminate BC patients from healthy subjects with 96% specificity, 98% sensitivity, and 97% accuracy (117, 118) while the combination of miR-142-5p and miR-320a could distinguish luminal A subtype from healthy individuals with 100% sensitivity, 93.80% specificity, and AUC 0.94 (117). The abovementioned information indicates that c-miRNAs found in various body fluids are strongly correlated with the health state and may serve as attractive biomarker candidates for the diagnosis and prognosis of BC (116) (**Figure 3**). Broad information on c-miRNAs with a significant role in BC is combined in **Table 3**. Apparently, the technique of detecting miRNAs for liquid biopsy is based on the following: after obtaining the liquid sample of a BC patient, total RNA is extracted, cDNA is synthesized, and RT-qPCR is performed to detect the relative expression of target miRNAs.

**Table 3:** Summary of recent studies since 2015 on altered levels of circulating miRNAs in breast cancer.

First author	Year of publication	Source/sample	Detection method	Number of subjectsor samples		AUC	miRNA	Levels	Main finding	Ref.
				BC patients	HC patients					
Zou et al.	2022	Serum	RT-qPCR	106	183	0.915	miR-133a-3p miR-497-5p mir-24-3p miR-125b-5p	↑	30 miRNAs were found to be dysregulated in BC. This suggests the promising potential of this robust and non-invasive prediction model for BC screening	(8)
							miR-377-3p miR-374c-5p miR-324-5p miR-19b-3p	↓		
Liu et al.	2022	Serum	RT-qPCR	112	59	0.697	miR-103-3p	↑	miR-103a-3p seems to be a non-invasive diagnostic and prognostic biomarker for BC	(86)
Gahlawat et al.	2022	Plasma	Qubit miRNA Assay Kit Qubit Fluorometer 3.0	250	N/A	N/A	Total cf-miRNAs	N/A	Measurement of total cf-miRNA level can be used as an LB marker for prediction of BC relapse and survival	(119)
Adam-Artigues et al.	2021	Plasma	RT-qPCR	54	89	0.77 0.92	miR-30b-5p miR-99a-5p	↑	The proposed two miRNAs seem to be promising candidates as non-invasive biomarkers for BC early diagnosis and prognosis improvement	(120)
				74	74					
Bao et al.	2021	Plasma EV	RT-qPCR	20	10	0.88 0.73 0.70	miR-421 miR-128-1 miR128-2	↑	Identified GI-derived three miRNA signatures (miR-421, miR-128-1, and miR-128-2) in the serum extracellular vesicles allows BC early detection	(121)
Chen et al.	2021	Plasma exosome	Molecular beacon assay	33	37	0.982	miR-1246	↑	Using mpsMB-1246 allows to directly measure miR-1246 for BC diagnosis without extracting plasma exosomes and their content	(122)
Diansyah et al.	2021	Plasma	RT-qPCR	26	16	N/A	miR-21	↑	Detection of c-miR-21 expression seems to have a potential diagnostic value for early-stage BC detection	(123)
Figueira et al.	2021	Plasma	RT-qPCR	N/A	N/A	N/A	miR-194-5p	↓	miR-194-5p and miR-205-5p as well as EVs seem to be promising biomarker candidates in early and advanced stages of BCBM	(124)
							miR-205-5p	↑		
Garrido-Cano et al.	2021	Plasma	NAA RT-qPCR	34	47	0.78	miR-99a-5p	↑	An NAA-based biosensor is a promising strategy to diagnose BC *via* miR-99a-5p detection in plasma	(125)
Jang et al.	2021	Plasma	RT-qPCR	226	146	0.992	miR-1246 miR-206 miR-24 miR-373	↓	Multiple miRNAs can serve as potential biomarker candidates for early diagnosis of BC	(118)
Jusoh et al.	2021	Plasma	PCR	8	9	> 0.7	miR-27b-3p miR-22-5p miR-145-5p	↑	miR-27b-3p, miR-22-5p, miR-145-5p can be used as potential biomarkers for detecting BC	(126)
Kim et al.	2021	EVs	RT-qPCR	62	20	0.90 0.86 0.88 0.84	miR-9 miR-16 miR-21 miR-429	↑	The combination of miR-9, miR-16, miR-21, and miR-429 from the EV can serve as sensitive and specific biomarkers for the early diagnosis of BC through LB	(127)
Liu et al.	2021	Serum Exosome	RT-qPCR	224	113	0.68	miR-423-5p	↑	hsa-miR-423-5p can be used as a non-invasive BC biomarker	(128)
Dwedar et al.	2021	Serum	RT-qPCR	61	48	0.98	miR-10b	↑	C-miR-10b seems to be a potentially non-invasive serum biomarker for BC diagnosis and prognosis	(129)
Simón et al.	2021	Plasma	RT-qPCR	38	40	0.89	miR-30b-5p	↑	miR-30b-5p expression levels can be used as an early diagnostic BC biomarker as they demonstrated a good diagnostic potential	(130)
Xun et al.	2021	Serum	RT-qPCR	N/A	N/A	N/A	miR-138-5p	↑	Exosomal miR-138-5p appears to be a promising prognostic BC biomarker	(131)
Liu et al.	2021	Bivariate meta-analysis	21 studies	N/A	N/A	0.92	miR-155	N/A	miR-155 has the capacity to facilitate accurate BC detection, thus, has the potential to be used as BC diagnostic biomarker	(132)
Ahmed et al.	2021	Plasma	RT-qPCR	57	20	0.826 0.721	miR-181b-5p miR-222-3p	↑	Circulating sEV-derived miR-181b-5p, miR-222-3p, and let-7a-5p appear to be promising non-invasive IBC diagnostic biomarkers	(133)
						0.918	let-7a-5p	↓		
Zhang et al.	2021	Whole blood	RT-PCR	68	13	0.957	miR-185-5p miR-362-5p	↑	Six identified upregulated miRNAs and a two-miRNA (miR-185-5p and miR-362-5p) panel extracted from the blood of BC patients may serve as potential biomarkers for BC diagnosis and prognosis	(87)
Wang et al.	2021	Plasma exosomes	RT-qPCR	10	10	0.733–0.958	miR-363-5p	↓	Exosomal miR-363-5p may be served as a biomarker used in LB strategies for LNM diagnosis in BC	(134)
Bakr et al.	2021	Serum	qPCR	196/76	49	0.98	miR-373	↑	Results prove that miR-373, as an oncomiR, would be a vital biomarker for BC diagnosis and prognosis by targeting both VEGF and cyclin D1	(135)
Nashtahosseini et al.	2021	Serum	RT-qPCR	40	40	0.774	miR-660-5p	↑	Results show a reasonable diagnostic accuracy of these microRNAs for the detection of BC	(89)
						0.716	miR-210-3p			
Hashimoto et al.	2021	Serum	miRNA chip RT-qPCR	14/21	6/9	0.99/ 0.98	miR-1307-3p	↑	miR-1307-3p appears to be a promising biomarker of diagnostic value for thirteen cancer types	(136)
Mohmmed et al.	2021	Serum	RT-qPCR	50	30	0.947	miR-106a	↑	miR-106a gene may serve as a potential genetic non-invasive biomarker in BC patients through regulating RAF-1 expression	(137)
Qattan et al.	2021	Blood	RT-PCR	93	34	0.65 0.65 0.63 0.63	miR-19a-3p miR-19b-3p miR-93-5p miR-210-3p	↑	Upregulated levels of miR-93, miR-210, miR-19a, and miR-19b are associated with poor overall survival outcomes in TNBC patients	(138)
Sadeghi et al.	2021	Blood	RT-PCR	70	60	0.82	miR-106b-5p miR-126-3p miR-140-3p miR-193a-5p miR-10b-5p	N/A	A novel multi-marker panel of miRNA seems to be a promising biomarker for detecting benign BC	(115)
Uyisenga et al.	2021	Plasma	RT-qPCR	143/82	136/73	> 0.8	miR-16-5p let-7d-5p miR-103a-3p miR-107 miR-148a-3p let-7i-5p miR-19b-3p miR-22-5p	N/A	A biomarker signature of 8 microRNAs can be measured in the plasma—via non-invasive and simple procedure	(139)
Zou et al.	2021	Blood	RT-qPCR	538	100	0.774 0.881	miR-451a miR-195-5p miR-126-5p miR-423-3p miR-192-5p miR-17-5p	N/A	The identified circulating six-miRNA biomarker panel can be used for differentiation of benign and malignant breast lesions along with mammography	(140)
Ashirbekov et al.	2020	Plasma	qPCR	35	33	0.984	miR-145-5p miR-191-5p	↑	Two plasma miRNAs: miR-145-5p and miR-191-5p appear to serve as potential biomarkers for BC diagnosis in the Kazakh population.	(141)
Yadi et al.	2020	Blood	RT-qPCR	20	N/A	N/A	miR-4638-3p miR-1273 g-3p	N/A	miR-4638-3p and miR-1273 g-3p play a pivotal role in cardiotoxicity induced by anthracyclines in BC patients	(142)
Holubekova et al.	2020	Plasma	RT-qPCR	65	34	N/A	miR-99a miR-130a miR-484 miR-1260a	↑	Identified miR-99a, miR-130a, miR-484, and miR-1260a are significantly upregulated in the plasma of BC patients	(143)
Ibrahim et al.	2020	Plasma	RT-qPCR	30	20	≥ 0.7	miR-21 miR-181a miR-10b	↑	OncomiRs miR-10b and miR-21 may serve as promising biomarkers for the prediction of progression-free survival	(144)
							miR-145 let-7a	↓		
Koi et al.	2020	Serum	Agilent 2100 Bioanalyzer	78	72	0.92	miR-21-5p miR-23a-3p	↑	Identified miRNAs appear to be promising BC detection biomarkers	(145)
Ahmed et al.	2020	Serum	RT-qPCR	44	19	0.895 0.965	miR-29a miR-335	↓	Downregulated expression levels of miR-29a and MiR-335 can be considered the indicator of BC metastasis development	(146)
Garrido-Cano et al.	2020	Plasma	RT-qPCR	105	98	0.7555	miR-99a-5p	↑	miR-99a-5p can be taken as a promising non-invasive biomarker for the detection of BC	(147)
García-Magallanes et al.	2020	Serum	RT-qPCR	27/17	N/A	N/A	miR-145-5p miR-133a-3p	↓	miR-145-5p and miR-133a-3p may be considered tumor suppressors. miR-145-5p may serve as a biomarker for the BC diagnoses while miR-133a-3p may be used for the classification of BC	(148)
Anwar et al.	2020	Plasma	RT-qPCR	102	15	N/A	miR-155	↑	C-miR-155 can be used as the diagnostic marker in BC	(149)
Aksan et al.	2020	Serum	RT-qPCR	45	48	0.806 0.658	miR-21	↑	Serum miR-21 may be used as a non-invasive biomarker that can aid in the differentiation of IGM from BC	(150)
							let-7c miR-155	↓		
Wang et al.	2020	Serum	RT-qPCR	45	50	0.894	miR-188-5p	↑	miR-188-5p is a tumor suppressor miRNA in BC progression and may be used as a non-invasive diagnostic biomarker as well as BC therapeutic target	(151)
Kim et al.	2020	Plasma	RT-qPCR	30	30	0.95	miR-202	↑	C-miR-202 might serve as a potential biomarker for early-stage BC detection	(152)
Zou et al.	2020	Plasma Serum	RT-qPCR	354	404	0.650-0.757	miR-188-3p miR-500a-5p miR-501-5p miR-188-3p miR-501-3p miR-502-3p miR-532-3p miR-532-5p	↑	The identified miRNAs seem to be promising non-invasive biomarkers for BC diagnosis	(153)
Wang et al.	2020	Serum exosome	RT-PCR	55	55	0.886	miR-1910-3p	↑	MiR-1910-3p extracted from serum exosomes may be considered a novel molecular marker for the diagnosis of BC	(154)
Moln et al.	2020	Plasma	qPCR	4/5	N/A	N/A	miR-802 miR-194	↓	Downregulated levels of c-miR-802-5p and miR-194-5p seem to be a precocious event in BCBM while MEF2C–a target of both miRNAs plays a significant role in the development of brain metastasis	(155)
Yan et al.	2019	Plasma EV	PCR	12	10	0.785 0.808	miR-24-2-5p miR-205-5p miR-375	↑	miRNA- 548b-5p and miRNA- 376b-5p are positively associated with patient survival while miRNA- 375 and miRNA- 24-2-5p are negatively associated with patient survival that may serve as biomarkers for early-stage BC diagnosis	(156)
							miR-376b-5p miR-548b-5p miR-655-3p	↓		
Souza et al.	2019	Serum	NanoString nCounter System	54	24	0.74	miR-25-3p	↑	miR-25-3p can distinguish patients with TNBC from healthy controls. LB approach using molecular biomarkers can be used for BC screening with the potential of minimal invasiveness	(157)
Raheem et al.	2019	Serum	RT-qPCR	30/30	30	0.842	miR-34a	↓	Significantly decreased expression level of miRNA-34a is observed in the serum of BC patients, hence, miR-34a can be employed as a potential non-invasive molecular marker for the BC early diagnosis	(158)
Shiino et al.	2019	Serum	Microarray	958	N/A	0.86	miR-629-3p	↑	Serum miRNA profiles may be served as a minimally invasive biomarker for the diagnosis of ALN metastasis before surgery	(159)
							miR-4710	↓		
Li et al.	2019	Plasma exosomes	RT-qPCR	257	257	0.683 (tr.) 0.966 (test) 0.978 (val.)	Let-7b-5p miR-122-5p miR-146b-5p miR-210-3p miR-215-5p	↑	The 5-miRNA plasma panel (let-7b-5p, miR-122-5p, miR-146b-5p, miR-210-3p and miR-215-5p) seems to be a favorable biomarker for BC detection	(160)
Pereira et al.	2019	Blood, Plasma	RT-qPCR	20/25 82/93	N/A	0.831	miR-30b-5p	↑	miR-30b-5p is abnormally upregulated in metastatic BrC and may play a significant role in tumor dissemination	(161)
Anwar et al.	2019	Plasma	RT-qPCR	102	15	N/A	miR-21	↑	MiR-21 expression is found to be elevated in BC patients and might serve as a therapeutic monitoring marker	(90)
Rodríguez-martínez et al.	2019	Serum	RT-qPCR	53	8	0.777	miR-21 miR-105 miR-222	↑	Detecting exosomal miRNAs and CTCs *via* LB seems to be a promising approach for allowing improved diagnosis and prognosis of BC	(162)
Tan et al.	2019	Blood	RT-qPCR	16	8	0.752	miR-106	↑	Using CTC-specific miRNAs as new biomarkers will allow the further optimization of personalized therapy for BC	(163)
Swellam et al.	2019	Serum	RT-qPCR	100	20	0.955	miR-27a	↑	Detection of miR-27a expression levels seems to be a promising non-invasive molecular marker for BC early detection	(164)
Mcanena et al.	2019	Plasma	NGS	31	21	0.902	miR-331	↑	A higher expression levels of mir-331 and lower expression levels of mir-195 may be considered biomarkers for distinguishing metastatic and local BCs	(165)
							miR-195	↓ (ns)		
Saleh et al.	2019	Serum	RT-qPCR	90	60	0.957	miR-122	↑	C-miR-122 may be used as a diagnostic and prognostic biomarker in BC patients	(166)
Abdulhussain et al.	2019	Serum	RT-qPCR	60	30	1	miR-21	↑	The c-miR-21 level may serve as a marker for women’s BC early detection	(167)
Incoronato et al.	2019	Serum	RT-qPCR	77	78	0.85 0.80	miR-125b-5p miR-143-3p	↑	The two miRNAs are potential biomarkers for the prognosis of BC	(168)
Papadaki et al.	2019	Plasma	RT-qPCR	133/110	23	0.797	miR-21 miR-23b miR-190 miR-200b miR-200c	↑	A panel of four miRNAs can be used as biomarkers to differentiate early and MBC	(169)
Arabkari et al.	2019	Serum	RT-qPCR	38	20	0.875	miR-145 miR-486	↑	The panel of miR-145, miR-195, and miR-486 has the best diagnostic value for luminal A BC	(170)
							miR-195	↓		
Di Cosimo et al.	2019	Plasma	RT-qPCR	183/246	N/A	0.86	miR-140	↑	There is promising evidence that circulating miRNAs can discriminate the patients with and without the pathological complete response after lapatinib- and/or trastuzumab-based therapy	(171)
Swellam et al.	2019	Serum	RT-qPCR	106	40	0.971	miR-335	↓	Assessment of circulating miRNA expression level may serve as a minimally invasive marker for BC prediction and diagnosis	(172)
Yu et al.	2018	Serum	RT-qPCR	113	47	0.895	miR-21-3p miR-21-5p	↑	A panel of three miRNAs is identified as a prospective biomarker for the early detection of BC	(173)
							miR-99a-5p	↓		
Li et al.	2018	Plasma	RT-qPCR	200	200	0.822 0.658 0.825 0.624	miR-106a-3p miR-106a-5p miR-20b-5p miR-92a-2-5p	↓	The identifie plasma and serum miRNAs from miR-106a–363 cluster may be considered favorable novel biomarkers for BC diagnosis	(174)
		Serum	RT-qPCR	204	202	0.914 0.913 0.915 0.829	miR-106a-5p miR-19b-3p miR-20b-5p miR-92a-3p			
Zhang et al.	2018	Meta-analysis	RT-qPCR	N/A	N/A	N/A	miR-20a	↓	Change in circulating and tissue-based miR-20a expression levels has an essential prognostic implication for human cancers	(175)
Hesari et al.	2018	Serum	RT-qPCR	100	142	N/A	miR-17	↑	Upregulation of miR-17 and downregulation of miR-25 and miR-133 in the patient’s serum are correlated with BC	(176)
							miR-25 miR-133	↓		
Hu et al.	2018	Serum	TIRFM	23	29	N/A	miR-21	↑	miR-21 is upregulated in BC samples while miR-16 shows no difference in healthy and BC patient samples	(177)
Zhai et al.	2018	Plasma exosome	Nanoflare probe	46	28	0.982	miR-1246	↑	A study has developed a BC diagnostic assay that uses the gold nanoflare probe to detect the plasma exosomal miR-1246 expression level as a BC diagnostic biomarker	(178)
Niedźwiecki et al.	2018	Serum	RT-qPCR	46	N/A	N/A	miR-200c	↓	The level of miR-200c was lower in TNBC patients compared with the ER/PR positive group that makes it a promising potential biomarker	(179)
Ali et al.	2018	Serum	RT-qPCR	60	20	0.710 0.750	miR-182 miR-375	↑	miRNAs 182 and 375 can serve as potential non-invasive markers used for screening BC patients	(180)
Wang et al.	2018	Serum	Microarray	24 (tr.) 58 (val.)	24 (tr.) 44 (val.)	0.845 0.930	miR-222-3p	↑	miR-130b-5p, miR-151a-5p, miR-206, and miR-222-3p may may be considered promising biomarker candidates for BC diagnosis and prognosis	(181)
Zhu et al.	2018	Serum	TaqMan miRNA microarray RT-qPCR	109	N/A	0.594- 0.800	miR-222 miR-20a	↑	Dynamics of c-miRNAs might help predict clinical response to NCT in BC patients	(182)
							miR-451	↓		
Masuda et al.	2018	Serum	Microarray RT-qPCR	330	N/A	N/A	miR-488	↑	Circulating pre-miR-488 expression could serve as a novel prognostic biomarker for recurrence prediction in BC patients	(183)
Guo et al.	2018	Serum	Microarray	194	100	0.881 0.778	miR-1915-3p	↑	miR-1915-3p might play a role in BC development while serum miR-1915-3p and miR-455-3p may serve as diagnostic and predictive biomarkers for BC	(184)
							miR-455-3p	↓		
Papadaki et al.	2018	Plasma	RT-qPCR	133	23	>0.61	miR-21 miR-23b miR-200c	↑	Results demonstrate that detecting the expression levels of c-miRNAs may serve as potential biomarkers in early BC	(185)
							miR-190	↓		
Fan et al.	2018	Serum	BRCA RT-qPCR	49	19	0.936 0.884 0.793 0.964	miR-16 miR-21 miR-155 miR-195	↑	Serum levels of c-miR-16, c-miR-21, c-miR-155, and c-miR-195 can be used as biomarkers for early identification of BC, and for distinguishing BC patients from healthy controls	(186)
Swellam et al.	2018	Serum	RT-qPCR	80	30	>0.8	miR-17-5p miR-155 miR-222	↑	Detection of the miR-17-5p, miR-155, and miR-222 expression levels in serum samples may serve as significant promising molecular markers for the diagnosis of early BC	(187)
Swellam et al.	2018	Serum	RT-qPCR	137	38	0.987	miR-373	↑	Detection of miRNAs in serum can be used as non-invasive biomarkers for BC early detection	(188)
Cui et al.	2018	Serum	NNC RT-qPCR	429	895	≥ 0.9	miR-1246 miR-6756-5p miR-8073	↑	A study constructed and validated an NNC-based biomarker panel consisting of three miRNAs for BC early detection	(189)
Alunni-fabbroni et al.	2018	Whole blood	RT-qPCR	48	17	≥ 0.69	miR-19a miR-22 miR-127 miR-20a miR-200b	N/A	Identification of whole blood miRNAs may allow to better distinguish post-operative EBC patients without any sign of metastasis to prevent later relapse	(190)
Elghoroury et al.	2018	Serum	RT-qPCR	75	50	N/A	miR-21	↑	Serum miR-21 in patients with BC serves as a novel non-invasive biomarker for BC detection while the association of let-7 with metastasis makes it a potential prognostic biomarker for patient stratification and treatment optimization	(191)
							let-7	↓		
Li et al.	2017	Plasma	RT-qPCR	118	12	0.657 0.928	miR-93-3p miR-105	↑	Circulating levels of miR-105/93-3p and miR-105 may serve as diagnostic biomarkers for TNBC	(192)
Qattan et al.	2017	Plasma	RT-qPCR	57	34	≥ 0.68 ≥ 0.67	miR-195-5p let-7	↑	miR-195 and let-7 seem to be satisfactory biomarker candidates	(193)
Jurkovicova et al.	2017	Plasma	RT-qPCR	137	28	0.745	miR-17 miR-27a	↓	miR-27a and miR-17 may be used as a potential diagnostic BC marker	(194)
Sueta et al.	2017	Exosome	RT-qPCR	35+39	N/A	N/A	miR-338-3p miR-340-5p miR-124-3p	↑	Several exosomal miRNAs may be useful biomarkers to predict the recurrence of BC	(195)
							miR-29b-3p miR-20b-5p miR-17-5p miR-130a-3p miR-18a-5p miR-195-5p miR-486-5p miR-93-5p	↓		
Zhang et al.	2017	Plasma	RT-qPCR	259	94	0.557 0.582	miR-200c miR-141	↑	Circulating levels of miR-200c and miR-141 seem to play an important role as biomarkers for the early detection of BC metastases	(196)
Gao et al.	2017	Plasma Exosome	TaqMan miRNA assay Nest-qPCR	259	94	0.77	miR-155	↑	Expression levels of plasma miR-155 may be used as a non-invasive biomarker for early-stage BC detection	(197)
Zeng et al.	2017	Plasma	RT-qPCR	173	75	N/A	miR-34a miR-34c	↓	Reduced miR-34a/c expression is strongly associated with tumor progression and indicated a worse prognosis	(198)
Zhang et al.	2017	Whole blood	miRCURY LNA™ array RT-qPCR	15	13	0.933 0.769 0.75 9 0.825 0.812	miR-30b-5p miR-96-5p miR-182-5p miR-374-5p miR-942-5p	↑	The identified five miRNAs may serve as novel biomarkers for BC detection and may be involved in the development and progression of BC	(199)
Shao et al.	2017	Meta-analysis	N/A	N/A	N/A	N/A	miR-203	↑	A high c-miR-203 expression level indicated poor prognosis in BC and colorectal cancer	(200)
Jinling et al.	2017	Meta-analysis	N/A	N/A	N/A	N/A	miR-21	↑	C-miR-21 expression level can be used for the prediction of poor prognosis in BC patients	(201)
Hamam et al.	2016	Serum Plasma	Microarray RT-qPCR	69	23	N/A	miR-4270 miR-1225-5p miR-188-5p miR-1202 miR-4281 miR-1207-5p miR-642b-3p miR-1290 miR-3141	↑	A novel approach was developed which led to the identification of a novel microRNA panel consisting of upregulated miRNAs in BC patients owing to the potential of serving as a diagnostic and stratification marker	(202)
Shimomura et al.	2016	Serum	Microarray RT-qPCR	1280	2836	0.971	miR-1246 miR-1307-3p miR-6861-5p	↑	A combination of miR-1246, miR-1307-3p, miR-4634, miR-6861-5p, and miR-6875-5p measured from serum can be employed for early-stage BC detection for differentiation of BC from pancreas, biliary tract, prostate benign diseases, or other cancers	(203)
							miR-4634 miR-6875-5p	↓		
Huo et al.	2016	Serum	RT-qPCR	44	31	0.65-0.86	miR-21-5p miR-194-5p miR-205-5p miR-375	↑	C-miRNAs are promising biomarkers as a minimally invasive multi-marker blood test for continuously monitoring BC recurrence	(204)
							miR-376c-3p miR-382-5p miR-411-5p	↓		
Mihelich et al.	2016	Serum	RT-qPCR	N/A	N/A	N/A	miR-182	↑	miR-182 was found to be a miR-183 family member that is trafficked by exosomes in the investigated cell types and human serum. In BC miR-182 may serve as a biomarker	(205)
Hamdi et al.	2016	Serum	RT-qPCR	20	20	N/A	miR-335	↑	miRNAs have potential to serve as diagnostic/prognostic biomarkers for IBC and non-IBC with links to the menopausal state, Her2 status, and parity	(206)
Matamala et al.	2015	Plasma	Microarray RT-qPCR	83/114	26/116	0.607-0.721	miR-21-5p miR-96-5p miR-125b-5p miR-505-5p	↑	Overexpression of miR-505–5p, miR-125b-5p, miR-21–5p, and miR-96–5p in plasma of BC patients are suggested to be used as non-invasive BC biomarkers	(207)
Li et al.	2015	Serum	RT-qPCR	90	64	0.848	let-7c	↓	let-7c serum levels of the BC patients are found to be significantly lower compared with the levels of the healthy controls making let-7c a promising biomarker candidate for BC diagnosis	(208)
Mangolini et al.	2015	Serum	ddPCR	28/59	27/35	≥ 0.66	miR-10b-5p	↑	The quantitative ddPCR approach for monitoring the absolute levels of specific miRNAs as diagnostic and prognostic serum biomarkers in BC patients is supported	(209)
							miR-148b-3p miR-652-3p	↓		
Sahlberg et al.	2015	Serum	RT-qPCR	33	30	0.701-0.810	miR-18b miR-103 miR-107 miR-652	↑	This signature seems to be a promising minimally invasive biomarker of tumor recurrence and overall survival for patients with TNBC	(210)
Ferracin et al.	2015	Serum, Plasma	ddPCR	18	18	0.665	miR-181a-5p	↑	The study suggests the use of cell-free miRNAs as cancer biomarkers and proposes miR-181a-5p as a promising diagnostic BC biomarker	(211)
Hagrass et al.	2015	Serum	RT-qPCR	120	50	N/A	miR-155 miR-195 miR-10b	↑	Systemic c-miRNAs can be potentially used as novel biomarkers for BC	(212)
Toraih et al.	2015	Serum	RT-qPCR	30	30	0.80 0.92 0.68	miR-21	↑	Expression level of serum miR-21 may be used as a promising non-invasive diagnostic/prognostic biomarker for BC	(213)
Zhang et al.	2015	Serum	Serum-direct Multiplex RT-qPCR	101	72	0.888 0.901	miR-199a miR-29c miR-424	↑	Serum-direct Multiplex-RT-PCR assay is an effective BC profiling method which uses small volumes and is compatible with Biobank	(214)
Zhang et al.	2015	Serum	RT-qPCR	58	93	0.87	miR-205	↓	Non-invasive miR-205 has a high clinical diagnostic value in the early-stage BC detection and plays an important role in the clinical diagnosis of various cancers	(215)
Shaker et al.	2015	Serum	RT-qPCR	80/20	30	0.996 0.993 0.978 0.993	miR-29b-2 miR-155 miR-197 miR-205	↑	miRNAs have great potential as biomarkers in BC	(216)

AUC, area under curve; cf-miRNA, cell-free microRNAs; RT-qPCR, reverse transcription-quantitative PCR; BC, breast cancer; NAA, nanoporous anodic alumina; EV, extracellular vesicles; GI, genomic instability; mpsMB, 2’-O-methyl and phosphorothioate modified MB; tr., training; val., validation; TNBC, triple-negative breast cancer; IGM, idiopathic granulomatous mastitis; BCBM, breast cancer brain metastasis; NGS, next generation sequencing; LB, liquid biopsy; sEV, small extracellular vesicle; IBC, inflammatory breast cancer; LNM, lymph node metastasis; MEF2C, myocyte enhancer factor 2C; CTC, circulating tumor cells; ns, not significant; MBC, metastatic breast cancer; ER, estrogen receptor; PR, progesterone receptor; NCT, neoadjuvant chemotherapy; EBC, early breast cancer; ALN, axillary lymph node. ↑, upregulated level of miRNAs; ↓, downregulated level of miRNAs; N/A, not applicable.

### miR-10b

miR-10b *via* binding to the mRNA of HOXD10 gene in 3’UTR inhibits its translation and gives rise to the upregulation levels of RHOC, therefore, invasion, and metastasis (38), as well as angiogenesis, are promoted in BC (48). Indeed, a number of studies have demonstrated that the levels of miR-10b are overexpressed in the serum of BC patients which makes this particular miRNA a promising target for developing a non-invasive and cost-efficient BC LB detection approach (129, 144, 209, 212). Ibrahim et al. have studied the expression levels of certain miRNAs in plasma during different steps– diagnosis, chemotherapy, and after the tumor resection in BC patients. According to their finding, it is suggested that the upregulation of miR-10b might be the diagnostic marker of BC (144). Mangolini et al. have also demonstrated the significance of miR-10b as a biomarker of tumor aggressiveness. Particularly, the study showed that serum levels of miR-10b-5p were increased significantly along with the tumor stage while there was no substantial difference observed between the miR-10b-5p expression levels in stage I and control subjects (209). Despite the evidence, there are more studies needed to strengthen the idea of miR-10b utility as a BC biomarker *via* the LB approach.

### miR-16

miR-16 is evidenced to be overexpressed in the blood of BC patients (127, 186). Indeed, Kim et al. have demonstrated the upregulation of miR-16 along with several more miRNAs in EVs of early-stage BC patients compared with the healthy subjects which indicates the involvement of miR-16 in tumor initiation. Their results suggested the promising outcome of the tested miRNA signatures for BC early diagnosis (127). On the contrary, Hu et al. showed no difference in the expression levels of miR-16 in BC patients and healthy subjects (177). The current evidence warrants the need for more and comprehensive studies on this miRNA to validate its role as a biomarker for LB in BC patients.

### miR-17

Despite a number of studies focusing on c-miR-17 expression regulation, there is no consistency in its expression levels in BC patients (176, 187, 194, 195). Hesari et al. have studied the changes in the levels of miR-17 in the serum of BC patients compared with control subjects and found that its upregulation is associated with augmented cell proliferation and poor survival time in patients (176). Swellam et al. have also revealed the concordance of the increased miR-17-5p serum levels and clinical stages that indicates its implication in cancer progression. Furthermore, the detection technique of miR-17-5p along with miR-155 and miR-222 with RT-PCR was superior to the tumor markers such as carcinoembryonic antigen (CEA) and Cancer antigen 15-3 (CA15.3) for BC early diagnosis (187). Conversely, Jurkovicova et al. have shown decreased levels of plasma miR-17 in BC patients compared with healthy controls (194). Hence, according to the current evidence, it is difficult to postulate whether this particular miRNA can serve as a biomarker candidate for the diagnosis and prognosis of BC.

### miR-20b

It is suggested that miR-20b plays a critical role in BC tumorigenesis. miR-20b is shown to have pro-angiogenic functions *via* inhibiting HIF-1A, STAT3 (49), and PTEN (50). Besides, it is also evidenced that its circulating levels are downregulated in BC patients (174, 195). This makes miR-20b-5p an interesting biomarker target for LB in BC.

### miR-21

Noteworthily, miR-21 is abundantly found oncomiR in BC and its augmented level is strongly associated with the poor prognosis of BC (38, 39). Based on recent studies miR-21 represents a key target for most scientists in terms of its dysregulated circulating levels in BC. Indeed, a number of studies have demonstrated that c-miR-21 levels are found to be significantly augmented in BC patients compared with the healthy controls (90, 123, 127, 144, 145, 150, 162, 167, 169, 173, 177, 185, 186, 191, 201, 204, 207, 213). Interestingly, both– miR-21-3p and miR-21-5p are found to be upregulated in BC (177). Moreover, miR-21 seems to promote tumor progression *via* inducing cell growth, invasion, and metastasis (40). Therefore, miR-21 is indeed suggested to be one of the most attractive candidates for LB approach development in BC. According to the abovementioned information miR-21 also represents a pro-angiogenic molecule (46). Indeed, the study demonstrated that the miR-21 knockdown with anti-miR-21 resulted in the suppression of angiogenesis and cell proliferation *via* inhibiting the HIF-1A/VEGF/VEGFR2-associated signaling pathway in mice with implanted 4T1 murine BC cells (44). Yu et al. have also validated the advantages of miR-21-3p and miR-21-5p as potential biomarkers for BC early detection (173). Similarly, Matamala et al. have hypothesized miR-21-5p as a biomarker candidate for non-invasive BC detection (207). Subsequently, miR-21 is the most studied oncomiR in BC and the results of various studies are consistent. Thus, miR-21 deserves more attention that may change the standards in BC detection and become the first approved c-miRNA-based biomarker for BC detection.

### miR-30b

The circulating levels of miR-30b are also noticed to be dysregulated in patients with BC. Particularly, its levels are reported to be augmented (120, 130, 161, 199). Indeed, Adam-Artigues et al. investigated the plasma levels of miR-30b and found it to be a valuable non-invasive diagnostic biomarker for BC. miR-30b may aid in the discrimination of stage I BC without clinical manifestation from the plasma samples which are obtained from healthy subjects (120). Zhang et al. have also found the upregulated miR-30b-5p to be a promising biomarker candidate for the diagnosis of BC in patients with very early-stage BC (199). Hence, miR-39b is suggested to be one of the candidates for LB approach development in BC detection, although, more studies are required for the complete validation of this hypothesis.

### miR-99a

c-miR-99a is reported to be significantly upregulated in BC patients (120, 125, 143, 147). However, Yu et al. identified a three-miRNA panel that might be a biomarker for early BC detection, and the levels of serum miR-99a-5p were found to be downregulated in BC (173) which causes discrepancy and requires validation with more studies before it is considered as a biomarker candidate for the diagnosis of BC *via* LB approach.

### miR-103a

miR-103a is suggested to be a biomarker for BC detection as its circulating levels are reported to be increased in patients with BC (86, 210). Liu et al. have investigated the circulating levels of miR-103a-3p in patients with BC and found them to be significantly upregulated when compared with the healthy subjects. Moreover, its expression levels in patients with positive Her2 status were higher than in subjects with Her2-negative status and patients with metastases exhibited higher levels of miR-103a-3p compared with the subjects without metastasis (86). Sahlberg et al. have suggested miR-103a-3p along with other miRNAs as a minimally invasive biomarker for tumor relapse in patients with TNBC (210). However, the studies are not sufficient enough until now and more cohorts are vital to validate its application in LB for BC detection and overall management.

### miR-125b

Despite the lack of studies on c-miR-125b-5p, there is no inconsistency in its levels in BC patients. It is reported that miR-125b-5p levels are upregulated in the plasma of BC (8, 168, 207). Incoronato et al. have investigated the BC-associated miRNAs and found miR-125b-5p to be upregulated in the plasma of BC patients and could distinguish BC patients from healthy controls (168). miR-125b-5p provides information about prognosis and, therefore, the potential use of this miRNA is suggested as a non-invasive BC biomarker after further validation with more studies is performed.

### miR-155

Evidently, miR-155 plays an important role in BC development. *Via* targeting the VHL gene it exhibits a pro-angiogenic role in BC tumorigeneses. miR-155 is shown to be upregulated in the blood of BC patients by a number of studies (149, 186, 187, 197, 212, 216). Anwar et al. studied the dynamic changes of c-miR-155 expression levels in BC patients and according to the results they concluded that women with BC expressed significantly higher levels of c-miR-155 compared with the healthy subjects while after the surgery and chemotherapy, its levels were found to be downregulated. Moreover, the expression levels were higher in patients with larger-size tumors (149). Fan et al. demonstrated that the serum level of c-miR-155 may serve as a biomarker for BC early diagnosis as well as discrimination of BC molecular subtypes (186). The same results were shown by Hagrass and colleagues who also concluded the potential of miR-155 utility as BC biomarkers (212). Kong et al. have demonstrated that except for promoting the angiogenesis, the upregulation of miR-155 promotes BC metastasis (35). Gao et al. suggested that the augmented levels of miR-155 seem to be associated with the initiation of the tumor but not the metastasis and that miR-155 may serve as a non-invasive biomarker for early-stage BC detection (197). Accordingly, miR-155 undoubtedly deserves further attention for its role in BC non-invasive detection *via* the LB technique.

### miR-200c

There is evidence demonstrating that circulating levels of miR-200 are augmented in BC patients (169, 185, 196), which makes it an attractive biomarker candidate for BC diagnosis/prognosis. Niedźwiecki et al. have investigated the serum levels of miR-200c and its association with TNCB. They compared the expression levels of c-miR-200c between TNBC and ER/PR-positive group and demonstrated that its circulating level was lower in patients with TNBC (179). As there are still discrepancies regarding its levels in BC patients, more studies are warranted for the validation of its utility as a biomarker molecule.

### miR-210

Interestingly, miR-210 targets the EFNA3 gene which makes it pro-angiogenic miRNA in BC tumorigenesis (54). Besides, a number of studies also demonstrate that the circulating levels of miR-210 are increased in BC patients (89, 138, 160). Qattan et al. have revealed that the significantly augmented levels of miR-210-3p which is implicated in certain signaling pathways are specific to the TNBC subtype of BC (138). The consistent results of the reported studies support the miR-210 utility as a promising biomarker molecule for the non-invasive detection of BC.

### miR-222

It has been observed that the levels of miR-222 are increased in the blood of patients with BC (133, 162, 181, 182). A study has revealed the significantly increased plasma levels of miR-222-3p in inflammatory BC patients (133). Similar results were shown in other studies (162, 181). Zhu et al. have observed the overexpression of miR-222 in BC and demonstrated its potential role as a predictive biomarker of the response to neoadjuvant chemotherapy in HR-positive/Her2-negative BC (182). Evidently, no controversy on the levels of this miRNA in different studies has been noticed. However, in order to strengthen this claim and make miR-222 a reliable biomarker candidate, more studies are required.

### miR-1246

Based on the evidence, miR-1246 is also suggested to exhibit a biomarker role in BC detection, thus, it may represent one of the key targets of LB development in BC. miR-1246 is shown to be upregulated in the blood of BC patients (122, 178, 189, 203). However, not all the studies are consistent (118). Thus, more cohorts are required to confirm the reliability of miR-1246 as a potential biomarker candidate for LB development in BC.

### let-7

miRNA let-7 (lethal-7) was discovered in C. elegans and, evidently, the abnormal expression levels and dysregulation of c-let-7 are observed in BC patients. Nevertheless, it is noteworthy that these studies are not consistent about the levels of c-let-7 in BC patients as some evidence its upregulation (193) while some show the downregulation (191). Elghoroury et al. have revealed that the downregulation of let-7 expression level is associated with the risk of metastasis in BC patients and, thus, let-7 is a promising biomarker of the BC prognosis and progression (191). Importantly, more studies have demonstrated its decreased levels (133, 144, 150, 191, 208) in the blood of BC patients which makes it an attractive biomarker candidate for LB.

## Challenges of liquid biopsy in breast cancer

Despite the number of advantages of LB in BC compared with tissue biopsy (217), some challenges of this approach still exist. E.g., because of the low concentration in the blood, the isolation and detection of CTCs are technically complicated which necessitate developing a specific method for it (75). In case of cfDNA application as a diagnostic biomarker, the major obstacle is the need for knowledge about tumor-specific variants (218). As to the ctDNA, its low concentration in the early stages of cancer makes the detection technically difficult while, on the other hand, the somatic mutations in blood stem cells become the source of the background noise and often lead to inaccurate interpretation of LB results (219). The main challenge for the application of c-miRNAs as biomarkers in BC diagnosis and prognosis is the detection and accurate quantification difficulties that are conditioned by their low abundance and small size in biofluids (220). Other than blood samples, there are insufficient studies on miRNAs detected in various biofluids (NAF, breast milk, saliva, urine, cervical fluid, etc.) for BC detection. Besides, more sensitive detection methods are needed for facilitating LB application in clinical settings. Additionally, the biology of each particular type of BC needs to be considered as different miRNAs exhibit different expression levels depending on the tumor types (116). This makes their use more challenging. Moreover, considering the huge number of miRNAs potentially and actually implicated in different BCs along with their complexity, there still is a lack of evidence to finally implement their utility as LB-detected biomarkers. Although a number of pre-clinical and clinical studies (**Table 4**) have been performed on c-miRNA dysregulation in BC for LB approach advancement, the most suitable miRNAs or miRNA panels that could be employed in clinics remain controversial. There are conflicting studies on certain c-miRNAs in BC, e.g., there is inconsistent evidence regarding the levels of miR-145. Some studies demonstrate its upregulated levels (126, 141, 170) while the others report its downregulation in BC (144, 148). This makes miR-145 a nonreliable target for the LB approach in BC diagnosis and prognosis. As well, miR-205-5p together with miR-194-5p is evidenced to exhibit a biomarker role in the detection of BC brain metastasis. miR-205-5p expression level is upregulated in 4T1-injected mice plasma compared with the control (124). However, there is no consistency in miR-205 levels in BC detection (215, 216). According to Zhang et al., miR-205 expression levels in the serum of control subjects are higher than in subjects with stage I and II (215) while Shaker et al. demonstrated significantly augmented serum levels of miR-205 in BC (216). Nevertheless, despite the mentioned shortcomings, single as well as panel miRNAs remain remarkably promising biomarker candidates for the early diagnosis and prognosis of BC.

**Table 4:** Clinical trials on breast cancer involving circulating miRNAs (sorted by study start date).

Clinical trial title	Location	Study type	Sponsor	Start Date	Number of participants	Status	ID
Circulating microRNA 21 expression level before and after neoadjuvant systemic therapy in breast carcinoma	Egypt	Observational	Ain Shams University	Dec. 2021	40	Not yet recruiting	NCT05151224
Aberrant expression of micro RNA for diagnosis of breast cancer	N/A	Observational	Assiut University	Dec. 2021	50	Not yet recruiting	NCT04720508
Interest of circulating tumor DNA in digestive and gynecologic/breast cancer	France	Interventional	Poitiers University Hospital	Jan. 2021	1000	Recruiting	NCT04530890
Adapted physical activity (APA) in a breast cancer population.	Italy	Interventional	University of Perugia	Jan. 2019	100	Active, not recruiting	NCT03528473
Early detection of cardiovascular changes after radiotherapy for breast cancer (EARLY-HEART)	FranceGermanyNetherlandsPortugalSpain	Interventional	Institut de Radioprotection et de Surete Nucleaire	Sep. 2017	250	Unknown	NCT03297346
miRNA and relevant biomarkers of BC patients undergoing neoadjuvant treatment	China	Observational	Cui Yimin	Nov. 2015	100	Unknown	NCT03779022
BreAst Cancer and Cardiotoxicity Induced by RAdioTherapy: the BACCARAT Study (BACCARAT)	France	Interventional	Sophie JACOB	Oct. 2015	120	Unknown	NCT02605512
The Andromeda Study.Predictive value of combined criteria to Tailor breast cancer screening.	Italy	Observational	Centro di Riferimento per l’Epidemiologia e la Prev. Oncologica Piemonte	Jul. 2015	26600	Completed	NCT02618538
REBECCA study (RadiothErapy for BrEast Cancer and CArdiotoxicity) (REBECCA)	France	Interventional	Sophie JACOB	Nov. 2014	0	Withdrawn	NCT02079272
Circulating microRNA as biomarker of cardiotoxicity in breast cancer	Poland	Observational	West Pomeranian Cancer Center	Jan. 2014	128	Completed	NCT02065908
IMaging PAtients for Cancer Drug selecTion - Metastatic Breast Cancer (IMPACT-MBC)	Netherlands	Interventional	University Medical Center Groningen	Aug. 2013	217	Active, not recruiting	NCT01957332
Identification and evaluation of biomarkers of resistance to neoadjuvant chemotherapy (IDEA SEIN) (IDEASEIN)	France	Interventional	Institut du Cancer de Montpellier-Val d’Aurelle	Jul. 2013	164	Completed	NCT03255486
Circulating miRNAs as biomarkers of hormone sensitivity in breast cancer	France	Interventional	Institut Claudius Regaud	Jun. 2012	39	Completed	NCT01612871
MicroRNA profiles in triple negative breast cancer (TARMAC)	Nigeria	Interventional	University College Hospital, Ibadan	Nov. 2021	42	Recruiting	NCT04771871
Circulating miRNAs	Ireland	Observational	Cancer Trials Ireland	May 2011	255	Completed	NCT01722851
STI.VI. Study: How to improve lifestyles in screening contexts (STIVI)	Italy	Interventional	Centro di Riferimento per l’Epidemiologia e la Prev. Oncologica Piemonte	May 2010	1270	Completed	NCT03118882

Searched on ClinicalTrials.gov database (April 2022) using “breast cancer” in condition’s bar and “circulating miRNA”, “circulating micro RNA”, or “circulating microRNA” in other term’s bar. N/A, not applicable.

## Summary

The current review aimed to collect the most relevant scientific findings regarding the LB technique in BC detection and discussed the potential attractive miRNAs for developing this non-invasive approach. In order to save time, minimize pain, increase efficiency, and overcome the rest of the obstacles which exist in the current screening methods, the development of LB for the early diagnosis of BC is critical. miRNAs, except for their function– post-transcriptional gene regulation, may also accomplish a biomarker role for the diagnosis and prognosis of BC *via* detecting their levels in various biofluids. Apparently, single miRNAs or miRNA panels have a great potential of improving the accuracy of clinical diagnosis and prognosis that will undoubtedly have a positive impact on BC management and treatment. Remarkably, some miRNAs including miR-21 and miR-155 have been demonstrated to play a major role in BC progression among other miRNAs which makes them special in BC management. Except for the evidence of increased blood levels of miR-21 and miR-155 in BC patients, it is shown that the urinary levels are also upregulated compared to healthy subjects. This makes these two miRNAs even more attractive targets for developing the LB approach in BC. Although a number of pre-clinical and observational/interventional clinical studies are focused on c-miRNAs for their utility for LB in BC, the consensus about the most suitable miRNA candidate or panel still remains a debate. Currently, there is no miRNA-based LB detection method employed in clinics yet. Therefore, in order to facilitate the development of the LB approach based on miRNAs in BC, more robust and well-designed studies including a large number of subjects are essential. Moreover, the utility of the LB approach is advantageous with its feasibility– minimal invasiveness, minimal pain, short procedure, simplicity, optimal for longitudinal monitoring, and cost-effectiveness. There is a considerable evidence that miRNA signatures may be employed successfully for LB in BC.

## Author contributions

The Authors DP, XLiu, and ZW contributed to the study conception, YL, XC, JL, ZhiL, LH, and ZheL contributed in data collection, DP and NR wrote the sections and prepared the original draft of the manuscript, DP worked on the visualization of the manuscript, NR reviewed and edited the manuscript, XLu and JM supervised. All authors contributed to manuscript revision, read, and approved the submitted version.

## Funding

This work was supported by the National Natural Science Foundation of China (82002820 and 82072740).

## Acknowledgments

Parts of the figures were created with BioRender.com.

## Conflict of interest

The authors declare that the research was conducted in the absence of any commercial or financial relationships that could be construed as a potential conflict of interest.

## Publisher’s note

All claims expressed in this article are solely those of the authors and do not necessarily represent those of their affiliated organizations, or those of the publisher, the editors and the reviewers. Any product that may be evaluated in this article, or claim that may be made by its manufacturer, is not guaranteed or endorsed by the publisher.
